# Efficacy and Safety of Microwave Ablation in Patients With Hepatocellular Carcinoma With Decompensated Liver Cirrhosis: A Retrospective Study

**DOI:** 10.1155/cjgh/4859487

**Published:** 2025-11-12

**Authors:** Jihua Xue, Yuting Gu, Ji Li, Junfei Zhang, Tingting Bian, Zhongsong Zhou, Yufeng Gao

**Affiliations:** ^1^ Department of Infectious Diseases, The First Affiliated Hospital of Anhui Medical University, Hefei, 230022, Anhui, China, ahmu.edu.cn; ^2^ Anhui Province Key Laboratory of Infectious Diseases, Anhui Medical University, Hefei, 230022, Anhui, China, ahmu.edu.cn

**Keywords:** decompensated, hepatocellular carcinoma, liver cirrhosis, microwave ablation

## Abstract

**Objective:**

Except for liver transplantation, no definitive treatment exists for hepatocellular carcinoma (HCC) in individuals suffering from decompensated liver cirrhosis. This research evaluated the feasibility and outcomes of microwave ablation (MWA) treatment in this patient population.

**Methods:**

This research involved individuals diagnosed with HCC and decompensated cirrhosis who underwent MWA between 2019 and 2022. The analysis examined complications associated with the procedure, the effectiveness of treatment, patient survival rates, and the variations in blood test results and liver function reserves before and after MWA.

**Results:**

The 62 enrolled patients were predominantly male (*n* = 48), and the average age was 59.06 years. Fifty‐one patients were diagnosed with HBV‐related cirrhosis. The tumor characteristics varied: 47 patients had single lesions with diameters ranging from 8 to 57 mm. Following the MWA procedure, there was a notable rise in the levels of alanine transaminase, aspartate transaminase, total bilirubin, prothrombin time, and the Child–Pugh, albumin–bilirubin, and model for end‐stage liver disease scores (*p* < 0.05). Analysis of survival data indicated that tumor diameter < 3 cm, complete response (CR), meeting the Milan criteria, and Barcelona Clinic Liver Cancer (BCLC) Stage 0‐A were linked to a more favorable prognosis. Multivariable Cox regression analysis identified achieving a CR as the strongest independent predictor for improved overall survival (HR = 0.25, 95% CI [0.09, 0.66], *p* = 0.005). Furthermore, the Milan criteria were independently associated with reduced risk of tumor progression in both univariate and multivariate analyses.

**Conclusion:**

MWA is a safe procedure for individuals with HCC and decompensated liver cirrhosis. CR is associated with a better prognosis.

## 1. Introduction

According to Global Cancer Incidence, Mortality, and Prevalence (GLOBOCAN) 2022 data, primary liver cancer ranks as the fourth most prevalent cancer and stands as the second leading cause of cancer‐related deaths in China [1]. Hepatocellular carcinoma (HCC) represents 70%–85% of all primary liver cancer instances, with over 80% of HCC cases occurring in individuals who have cirrhosis [2, 3]. Cirrhosis is a crucial step in the viral pathway involved in HCC development. Some patients already exhibit decompensated cirrhosis at diagnosis. The prognosis of these patients may be more closely linked to their overall liver function than to the specific tumor stage [4]. Therefore, assessing the hepatic functional reserve before selecting HCC treatment is crucial to minimize the risk of treatment‐induced worsening of liver function.

Real‐world data are important, an increasing number of treatment alternatives for HCC exist for patients with compensated cirrhosis. The guidelines for primary liver cancer indicate that local ablation therapy, transarterial chemoembolization (TACE), or transarterial radioembolization (TARE) can bring benefits to patients with compensated cirrhosis [5, 6]. However, the options for managing HCC in decompensated cirrhosis are limited, often leaving liver transplantation as the only specific treatment [7, 8], which is restricted by criteria and donor shortages, highlighting the need for alternative treatments for advanced liver disease. Typically, patients in this group are thought to have lower survival rates and higher adverse events (AEs) even after systemic treatments. Information on the use of locoregional therapies in these patients is scarce. However, overall survival (OS) may be extended in carefully selected patients and with strict monitoring for AEs. For instance, in individuals suffering from decompensated cirrhosis and multinodular HCC, superselective TACE demonstrates better survival outcomes compared to palliative treatment [9, 10]. Our clinical experience suggests that locoregional therapies can significantly prolong survival in individuals suffering from decompensated liver cirrhosis.

This study investigated whether locoregional therapies offer survival benefits to this patient population. Local thermal ablation (LTA) is a safe and well‐established local therapy for HCC with minimal impact on liver function. In China, LTA has gained wide acceptance, with reported survival rates comparable with those of surgical removal for HCC that meets the Milan criteria [11]. Although several small retrospective studies have reported survival benefits of LTA in patients with poor liver function [10, 12–18], the results lack consistency, making treatment decisions challenging. Given the promising response rates associated with microwave ablation (MWA) [19], this study analyzed its therapeutic efficacy and safety in individuals suffering from HCC and decompensated cirrhosis treated at our center.

## 2. Materials and Methods

### 2.1. Study Design

This single‐arm, retrospective feasibility study was reviewed and approved by the Ethics Committee of the First Affiliated Hospital, Anhui Medical University (Hefei, China), with the approval number: PJ2022‐13‐41, dated November 10, 2022. Each participant gave their written consent to take part in the study and agreed to the publication of their data. We assessed the feasibility and outcomes of MWA in treating patients with HCC and decompensated cirrhosis. Treatment efficiency was assessed and follow‐up was performed after MWA. The primary outcome measures were OS and mortality. The secondary outcomes included local tumor progression (LTP), progression‐free survival (PFS), changes in hepatic dysfunction assessed using Child–Pugh (C‐P), albumin–bilirubin (ALBI), and model for end‐stage liver disease (MELD) scores, as well as AEs. LTP refers to the appearance of tumor foci at the edge of the ablation zone after at least 1 contrast‐enhanced follow‐up study has shown complete ablation. PFS is defined as the length of time from the initiation of treatment to the first occurrence of disease progression or death from any cause, whichever occurs first. An AE was defined as any event considered to endanger patient health or safety, graded according to CTCAE v5.0.

### 2.2. Patients

This study enrolled individuals diagnosed with HCC and decompensated cirrhosis, as confirmed by the presence of ascites, hepatic encephalopathy, and/or variceal bleeding [20, 21], between January 2019 and February 2022. All patients underwent MWA. The identification of HCC was determined by characteristic angiographic findings on contrast‐enhanced ultrasound (CEUS), contrast‐enhanced computed tomography (CT) scans, or contrast‐enhanced magnetic resonance imaging (MRI). The inclusion criteria were as follows: no signs of metastasis outside the liver or invasion of the portal vein. Ensure that the patient has no active gastrointestinal bleeding and hepatic encephalopathy of Grade II or above during ablation through drug treatment, thus avoiding the patient being unable to cooperate with the surgery due to shock or consciousness disorders. Diuretic therapy is initiated when ascites affects respiratory function to enable the patient to tolerate the procedure.

### 2.3. MWA Procedure

MWA was performed under ultrasound guidance by two sonographers with 10 years of experience in interventional therapy for HCC, respectively. The MWA protocol was planned based on imaging findings, including HCC size and infiltration range, obtained using CEUS or contrast‐enhanced MRI. A MWA therapy device (KY2000, Canyon Medical Inc.) was used with a power of 50–60 W at a frequency of 2450 MHz for 5–10 min at each site. A safe margin of at least 0.5–1.0 cm was maintained between the ablation zone and the tumor margin.

Patients received intravenous conscious sedation combined with local anesthesia and were placed supine with the right arm elevated to facilitate subcostal access. A 14–17G cooled‐tip antenna was placed into the tumor center under ultrasound guidance. Output power was adjusted appropriately for each case. The ablation zone was monitored using real‐time ultrasound, and ablation was concluded when the hyperechoic zone adequately covered the lesion. Immediately postablation, pulsed energy was used to cauterize the needle tract during withdrawal. Technical success was defined as absent arterial enhancement on postprocedural contrast imaging.

### 2.4. Assessment of Treatment Efficiency and Follow‐Up

All patients were followed up until death or October 2023. CEUS was performed immediately after MWA to identify any residual tumor tissue. Liver function was assessed 3 days after MWA. All patients underwent contrast‐enhanced MRI 1 month after MWA. Early tumor necrosis was defined as the absence of enhancement within the treated area, which encompassed the original tumor and well‐defined margins. Subsequently, the regular follow‐ups included laboratory tests and contrast‐enhanced MRI at 3‐month intervals. Two radiologists who have over a decade of experience in interpreting liver scans, respectively, reviewed all follow‐up MRI studies. Assessment of efficacy was performed using mRECIST criteria. Complete response (CR) was defined as the disappearance of arterial enhancement in all target lesions (indicating no viable tumor imaging) and the absence of new lesions. Incomplete response includes partial response and stable disease. Partial response was defined as a ≥ 30% reduction in the sum of the diameters of the viable portions of target lesions (compared to baseline) without the appearance of new lesions. Stable disease was defined as changes in target lesions that did not meet the criteria for partial response.

### 2.5. Statistical Analysis

Continuous data with normal and non‐normal distributions are presented as mean ± standard error (SE) and median (interquartile range [IQR] or range), respectively. Comparisons were made using the paired *t*‐test for normal data and the Wilcoxon signed‐rank test for non‐normal data. We estimated survival using the Kaplan–Meier method and compared survival curves between different groups using the log‐rank test. Exploratory subgroup analyses were conducted based on age, sex, tumor number, tumor diameter, Milan criteria, Barcelona Clinic Liver Cancer (BCLC) stage, TNM stage, cirrhosis cause, hepatitis B virus (HBV) DNA level, CR status, pre‐MWA alpha‐fetoprotein (AFP) level, and pre‐MWA liver function reserve. To evaluate the independent prognostic value of clinicopathological factors on OS, univariate Cox regression was initially conducted. Variables significantly associated with the outcome (*p* < 0.10) were included in the multivariate Cox model. The proportional hazard (PH) assumption was assessed globally and per variable using Schoenfeld residuals, with no material violations detected. Results are presented as adjusted hazard ratios (HRs) with 95% confidence intervals (CIs). Statistical analyses were conducted utilizing IBM SPSS Statistics for Windows, Version 24.0 (IBM Corps., Inc., Armonk NY, USA). The starting point of survival was defined as the first day after MWA for HCC treatment. A *p* value of less than 0.05 was deemed to be statistically significant.

## 3. Results

This study enrolled 62 patients (48 men, 14 women; age range 37–77 years; mean ± SE, 59.06 ± 1.15 years) with a body mass index (BMI) range of 16.23–37.11 (mean ± SE, 23.46 ± 0.47) kg/m^2^ between 2019 and 2022. Twenty patients had AFP levels > 400 ng/mL, whereas 42 patients had levels ≤ 400 ng/mL. All patients had cirrhosis (fifty‐one HBV‐related, three hepatitis C virus (HCV)–related, four alcoholic, one Budd–Chiari syndrome, and three unexplained). Among patients with HBV‐related cirrhosis (*n* = 51), 37 received anti‐HBV therapy, and two of the 3 with HCV‐related cirrhosis received anti‐HCV therapy (Table 1).

**Table 1:** Patient demographic and baseline characteristics (*n* = 62).

Variable	All patients
Age	
Mean, SE	59.06, 1.15
Sex‐no (%)	
Male	48 (77.4%)
Female	14 (22.6%)
BMI (kg/m^2^)	
Mean, SE	23.46,0.47
AFP, ng/mL‐no (%)	
> 400	20 (32.3%)
≤ 400	42 (67.7%)
Cirrhosis	62 (100%)
Etiology of cirrhosis‐no.(%)	
HBV	51 (82.3%)
HCV	3 (4.8%)
Alcohol	4 (6.5%)
Budd–Chiari syndrome	1 (1.6%)
Unknown cause	3 (4.8%)
Antiviral treatment‐no (%)	
Anti‐HBV treatment	37 (72.5%)
Anti‐HCV treatment	2 (66.7%)

*Note:* AFP, alpha‐fetoprotein.

Abbreviations: BMI, body mass index; HBV, hepatitis B virus; HCV, hepatitis C virus; SE, standard error.

The median C‐P score was 7 (IQR: 6–8), with the vast majority of patients classified as C‐P Class B (61/62, 98.4%) and only one patient (1.6%) in Class C. This was consistent with the ALBI grading system, where the median ALBI score was −1.84 (IQR: −2.48 to −1.84). Most patients were ALBI Grade 2 (50/62, 80.6%), with 5 patients (8.1%) in Grade 1 and 7 (11.3%) in Grade 3. Similarly, the median MELD score was 12 (IQR: 6–12), categorizing 52 patients (83.9%) as low risk, 8 (12.9%) as intermediate risk, and 2 (3.2%) as high risk. Ascites was common (58/62, 93.5%) and was managed with diuretics to a mild to moderate level prior to ablation. Overt hepatic encephalopathy was uncommon (3/62, 4.8%), and all cases were well‐controlled (Grade 0–1) on ammonia‐lowering therapy before ablation. The presence of varices was rare (1/62, 1.6%), and this patient received appropriate prophylactic therapy with no active bleeding (Table 2).

**Table 2:** Baseline liver function and comorbidity status (*n* = 62).

Variable	All patients
Child–Pugh scores (median, IQR)	7, 1
B (*n*)	61
C (*n*)	1
ALBI scores (median, IQR)	−1.84, 0.64
Grade 1 (*n*)	5
Grade 2 (*n*)	50
Grade 3 (*n*)	7
MELD scores (median, IQR)	12, 6
High risk	2
Intermediate risk	8
Low risk	52
Ascites (*n*)	58
Medications	Diuretic therapy
Control status	Mild to moderate ascites
Hepatic encephalopathy (*n*)	3
Medications	Ammonia‐lowering therapy
Control status	Grade 0‐1 HE
Varices (*n*)	1
Medications	Portal pressure‐ lowering and hemostatic therapy
Control status	No active bleeding

*Note:* IQR, interquartile range; ALBI, albumin–bilirubin.

Abbreviation: MELD, model for end‐stage liver disease.

Among the patients enrolled in this study, the number of lesions per patient ranged from one to three (47 with single lesions, five with two lesions, and 10 with three lesions). The largest and smallest lesions measured 57 mm and 8 mm, respectively, with a mean diameter of 31.85 ± 2.25 mm. Forty‐two patients met the Milan criteria, while 20 exceeded it. The BCLC staging distribution consisted of 14 cases with BCLC0, 36 cases with BCLCA, 11 cases with BCLCB, and 1 case with BCLCC. The TNM staging distribution was as follows: 14 (T1aN0M0), 31 (T1bN0M0), 16 (T2N0M0), 0 (T3N0M0), and one (T4N0M0). A total of 87 lesions were ablated. Liver biopsy was performed in 32 patients, revealing HCC and undetermined pathology in 29 and three patients, respectively (Table 3).

**Table 3:** Baseline characteristics of tumors (*n* = 62).

Variable	All patients
Tumor size (cm)	
Mean, SE	32.83, 2.39
Tumor numbers (*n*)	
Number of HCC lesions, 1/2/3/> 3	47/5/10/0
Milan criteria	42/20
TNM stages (*n*)	
T1aN0M0	14
T1bN0M0	31
T2N0M0	16
T3N0M0	0
T4N0M0	1
BCLC stage (*n*)	
BCLC0/A/B/C	14/36/11/1
Ablation numbers (*n*)	87
Liver biopsy (*n*)	
HCC/undetermined pathology	29/3

*Note:* HCC, hepatocellular carcinoma.

Abbreviations: BCLC, Barcelona Clinic Liver Cancer; SE, standard error.

Ablation therapy resulted in significant increases in alanine transaminase (ALT), aspartate transaminase (AST), total bilirubin (TBIL) levels, and prothrombin time (PT), as well as C‐P, ALBI, and MELD scores (all *p* < 0.05). Platelet (PLT) counts decreased significantly. While albumin (ALB) levels remained stable (Table 4). The treatment was well‐tolerated, with a low incidence of major complications. Abdominal infection occurred in 5 patients (8.1%), all of which (5/5, 100%) were graded as CTCAE Grade 3 and were effectively managed with anti‐infective treatment. Regarding oncologic outcomes, the CR rate was 59.68% (37/62). During the follow‐up period, 22 deaths (35.48%) were recorded. The mean OS duration was 21.92 months (SE, 1.56). The median follow‐up time for the entire cohort was 31 months (95% CI: 20.52–41.48). For PFS, the mean duration was 14.19 months (SE, 1.52), with a median PFS (mPFS) of 16 months (95% CI: 10.20–21.80). Among the patients who achieved a CR, LTP occurred in 4 cases (10.81%), with a mean LTP‐free time of 24.70 months (SE, 1.99). The 30‐day hepatic decompensation rate was 3.23% (2/62), with both cases presenting as worsened ascites, leading to 2 readmissions and 1 patient requiring paracentesis. The 90‐day hepatic decompensation rate was 11.29% (7/62). The manifestations included worsened ascites (5 cases), jaundice (1 case), and gastrointestinal bleeding (1 case). These events resulted in 7 readmissions, and 2 patients required paracentesis (Table 5).

**Table 4:** Comparison of liver function at baseline and posttreatment (*n* = 62).

Variable	Median (Q1, Q3)		*p*
	Pre‐MWA	Post‐MWA	
Child–Pugh score	7 (7, 8)	8 (7, 8)	0.023
ALBI score	−1.84 (‐2.19,‐1.55)	−1.81 (−2.11, −1.54)	0.029
MELD score	12 (9, 15)	14 (12, 17)	< 0.001
ALT (U/L)	30 (22, 40.50)	86.50 (55.75, 136)	< 0.001
AST (U/L)	36 (27.25, 53.50)	132 (83.25, 209)	< 0.001
TBIL (μmol/L)	25.25 (16.58, 37.35)	33.95 (25.23, 47.30)	< 0.001
PLT (× 10^9^/L)	61.50 (38.25, 95.5)	53 (39.25, 75.50)	< 0.001
PT (S)	16 (14.6, 17.95)	17.40 (15.45, 19.20)	< 0.001
ALB (g/L)	32.1 (29.23, 36.05)	32.9 (30.40, 35.88)	0.381

*Note:* ALT, alanine transaminase; AST, aspartate transaminase; TBIL, total bilirubin; ALB, albumin; PLT, platelet; ALBI, albumin–bilirubin.

Abbreviations: MELD, model for end‐stage liver disease; PT, prothrombin time.

**Table 5:** Treatment response, complications, and survival outcomes (*n* = 62).

Variable	All patients
Complications (abdominal infection)	5
CTCAE grade (Version 5.0) (*n*, %)	3, 100%
PAS	41
Complete response (*n*, %)	37, 59.68%
Death (*n*, %)	22, 35.48%
Survival duration (months)	
Mean, SE	21.92, 1.56
Median follow‐up (95% CI)	31 (20.52, 41.48)
PFS (months)	
Mean, SE	14.19, 1.52
mPFS	16 (10.20, 21.80)
LTP (*n*, %)	4, 10.81%
LTP‐free time	24.70, 1.99
30‐day hepatic decompensation (*n*, %)	2, 3.23%
Worsened ascites (*n*)	2
Readmissions (*n*)	2
Need for paracentesis (*n*)	1
90‐day hepatic decompensation (*n*, %)	7, 11.29%
Worsened ascites (*n*)	5
Jaundice	1
Gastrointestinal bleeding	1
Readmissions (*n*)	7
Need for paracentesis (*n*)	2

*Note:* mPFS, median progression‐free survival.

Abbreviations: LTP, local tumor progression; PAS, postablation syndrome; PFS, progression‐free survival; SE, standard error.

A total of 41 patients developed postablation syndrome (PAS) following MWA, with all experiencing fever. Specifically, three patients exhibited fever on the night of the MWA, while 37 others developed fever on the first day post‐MWA, and one patient experienced fever starting on the second day after MWA. The recorded body temperatures among these patients ranged from a minimum of 37.5°C to a maximum of 39.9°C. Additionally, six patients reported abdominal pain, which included discomfort in the surgical area and below the xiphoid process. Two patients presented with gastrointestinal symptoms, such as nausea, vomiting, and epigastric discomfort. All the AEs mentioned above were CTCAE Grade 1 and resolved with symptomatic management (Table 6).

**Table 6:** Characteristics of PAS (*n* = 41).

Variable	All patients
Fever (*n*)	41
Temperature range (°C)	37.5∼39
Time of fever onset (*n*)	
The first day after MWA	37
The day of MWA	3
The second day after MWA	1
CTCAE grade (Version 5.0) (*n*, %)	1, 100%
Pain (*n*)	6
Surgical site pain	1
Subxiphoid pain	2
Lumbosacral and abdominal pain	3
CTCAE grade (Version 5.0) (*n*, %)	1, 100%
Gastrointestinal symptoms	2
Nausea and vomiting	1
Gastric discomfort	1
CTCAE grade (Version 5.0) (*n*, %)	1, 100%

*Note:* MWA, microwave ablation.

Abbreviation: PAS, postablation syndrome.

**Figure 5:**
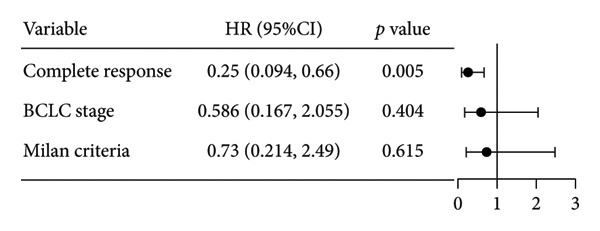
Forest plot of the impact of the clinical factors used in the univariate analysis on overall survival (OS). HR, hazards ratio; CI, confidence interval; BCLC, Barcelona Clinic Liver Cancer.

Survival analysis revealed no significant association between the prognosis and sex, age, BMI, etiology, tumor number, pre‐MWA AFP level, or pre‐MWA liver function reserve (C‐P, ALBI, and MELD scores) (Figures 1, 2, 3, and 4). Conversely, a smaller tumor diameter (< 3 cm), CR, within the Milan criteria, and BCLC0‐A stage were associated with an improved prognosis (Figure 3). The HRs and 95% CIs were as follows: HR = 3.31, 95% CI (1.35,8.11), *p* = 0.009; HR = 0.20, 95% CI (0.08,0.50), *p* < 0.001; HR = 3.00, 95%CI (1.30,6.91), *p* = 0.01; and HR = 2.97, 95% CI (1.20,7.35), *p* = 0.019. Multivariable Cox regression analysis revealed that CR was associated with the survival of patients with HCC and decompensated liver cirrhosis. Patients with CR had a 0.25‐fold higher risk of death than those with incomplete response (Figure 5). Additionally, the 30‐day landmark analysis, conducted to mitigate immortal time bias, demonstrated no significant difference in OS between the initial CR and nonresponse groups (Supporting Figure 1).

Figure 1Impacts of HBV infection and anti‐HBV treatment on overall survival (OS). (a) Comparison of HBV DNA levels between the anti‐HBV and non–anti‐HBV therapy groups; (b) Kaplan–Meier analysis of OS stratified by HBV status: positive vs. negative; (c) Kaplan–Meier analysis of OS stratified by HBV treatment: treated vs. untreated. HBV, hepatitis B virus; HBV DNA, hepatitis B virus deoxyribonucleic acid.

**Figure:**
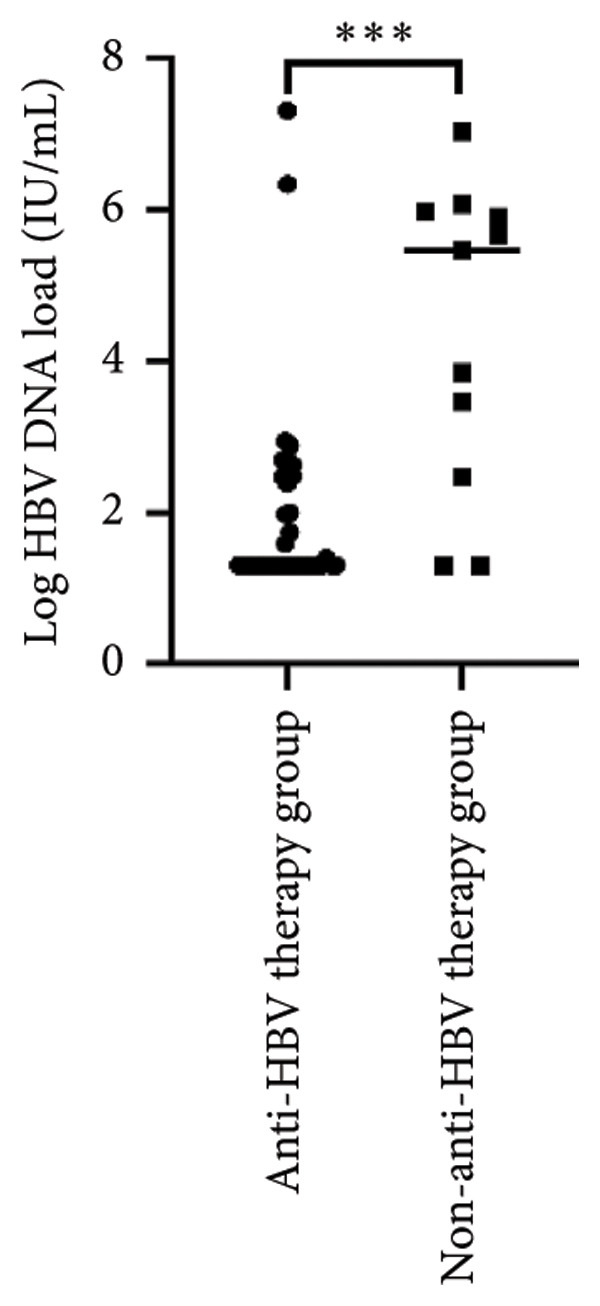
(a)

**Figure:**
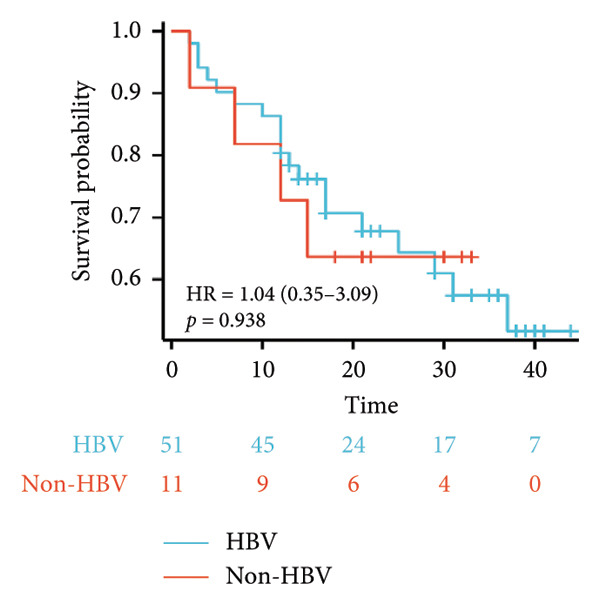
(b)

**Figure:**
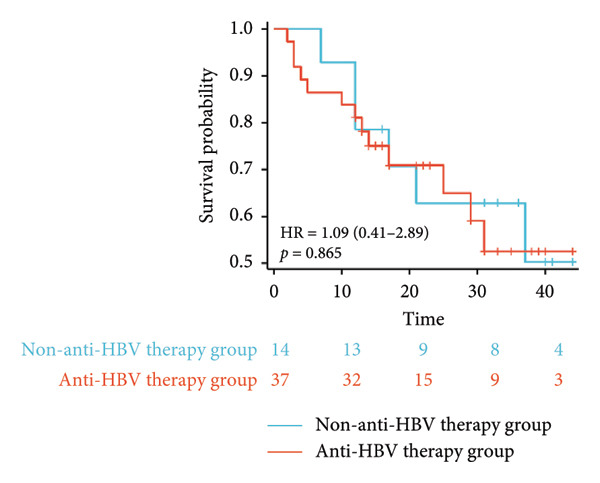
(c)

Figure 2Analysis of the impacts of patient characteristics on overall survival (OS). (a) Kaplan–Meier estimates of OS stratified by age: ≥ 60 vs. < 60 years; (b) Kaplan–Meier analysis of OS stratified by sex: male vs. female; (c) Kaplan–Meier analysis of OS stratified by AFP level: ≥ 400 vs. < 400 ng/mL; (d) Kaplan–Meier analysis of OS stratified by BMI: ≥ 24 kg/m^2^ vs. < 24 kg/m^2^. AFP, alpha‐fetoprotein; BMI, body mass index.

**Figure:**
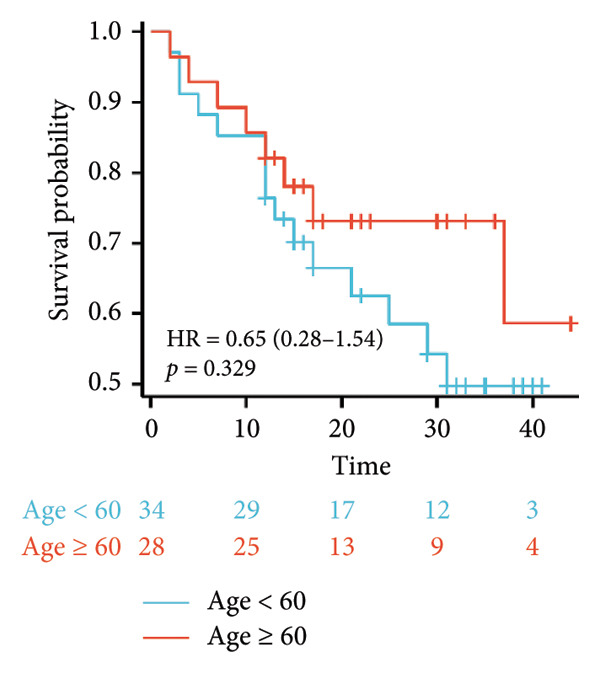
(a)

**Figure:**
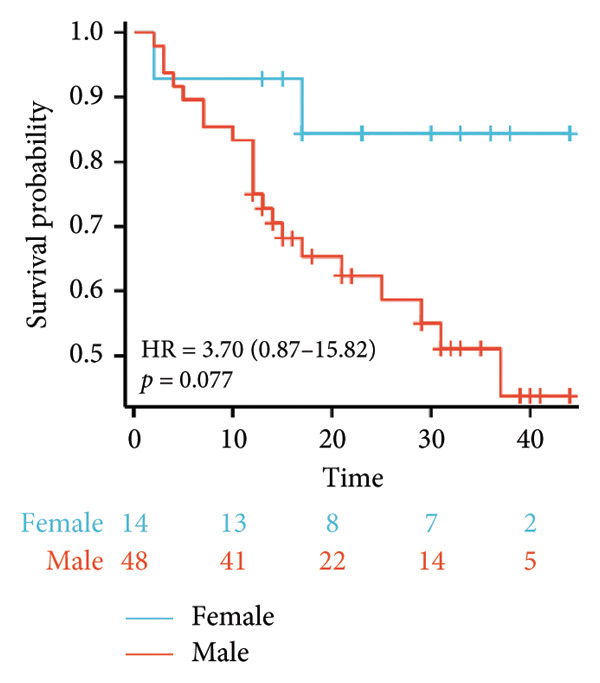
(b)

**Figure:**
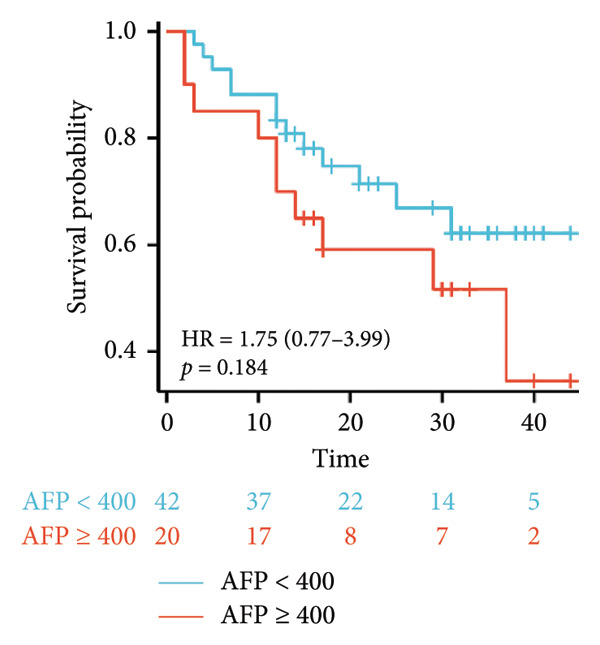
(c)

**Figure:**
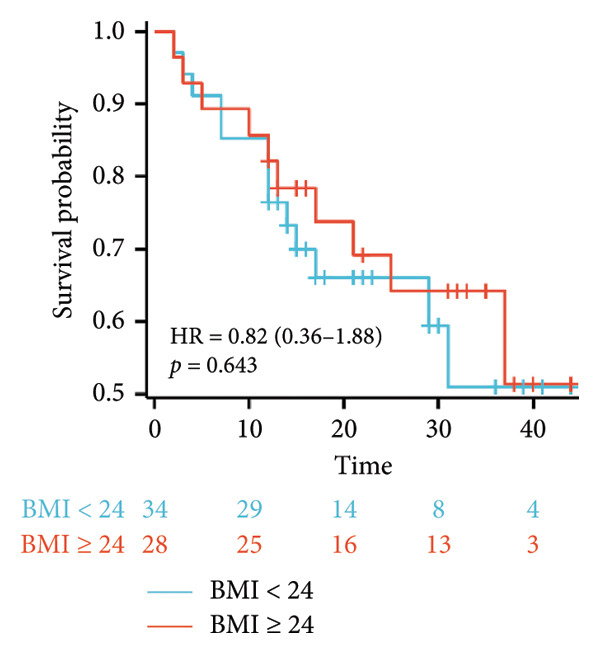
(d)

Figure 3Analysis of the impacts of tumor characteristics on overall survival (OS). (a) Kaplan–Meier analysis of OS stratified by tumor number: = 1 vs. > 1 tumor; (b) Kaplan–Meier analysis of OS stratified by treatment response: complete response vs. incomplete response; (c) Kaplan–Meier analysis of OS stratified by tumor diameter: < 3 vs. ≥ 3 cm; (d) Kaplan–Meier analysis of OS stratified by Milan criteria: within Milan criteria vs. beyond Milan criteria; (e) Kaplan–Meier analysis of OS stratified by BCLC stage: BCLC 0‐A vs. BCLC B‐C; (f) Kaplan–Meier analysis of OS stratified by TNM stage: T1a‐bN0M0 vs. T2‐4N0M0. BCLC, Barcelona clinic liver cancer; TNM, tumor lymph node metastasis.

**Figure:**
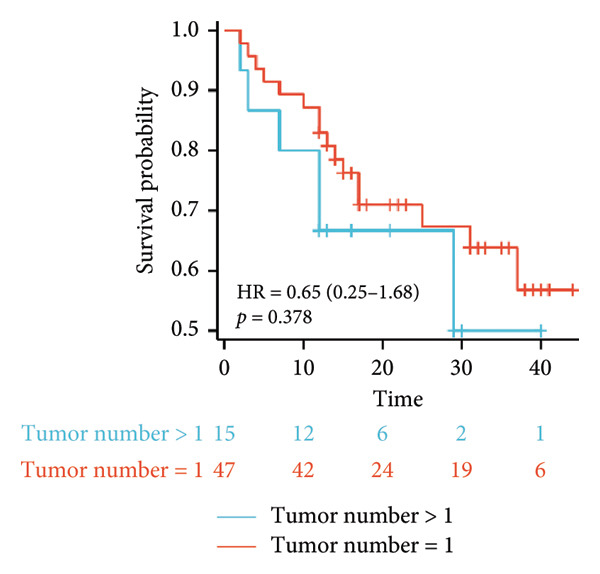
(a)

**Figure:**
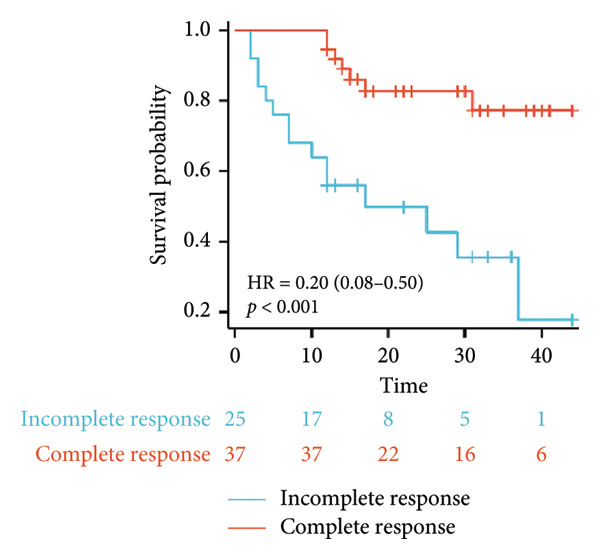
(b)

**Figure:**
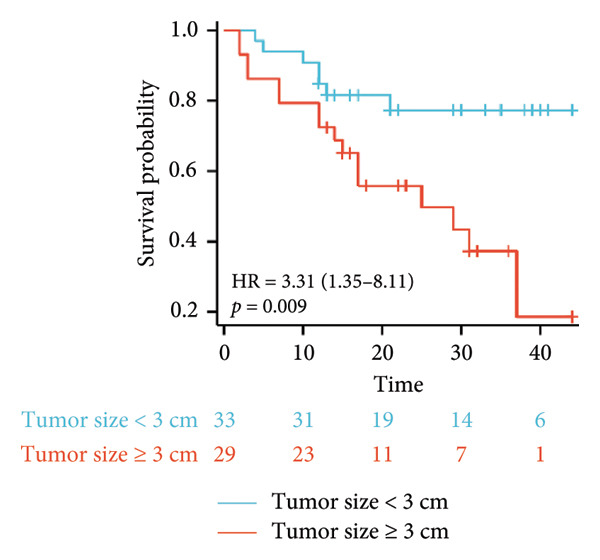
(c)

**Figure:**
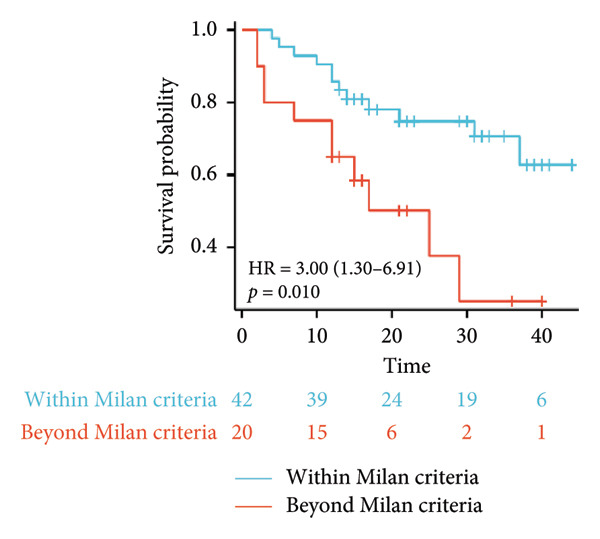
(d)

**Figure:**
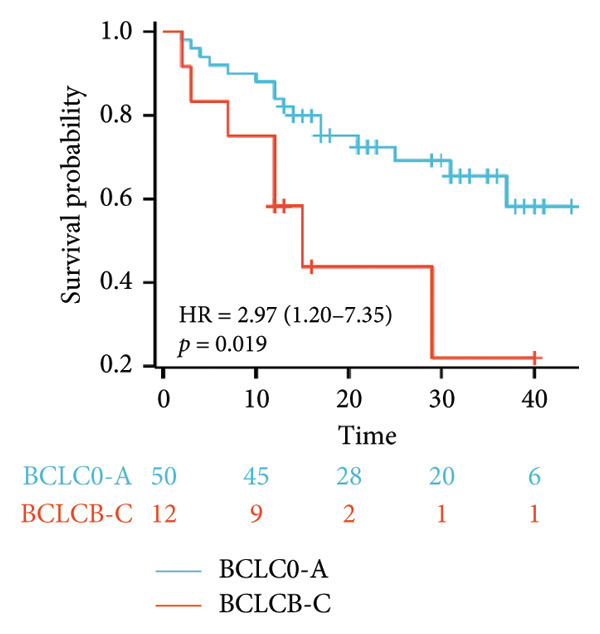
(e)

**Figure:**
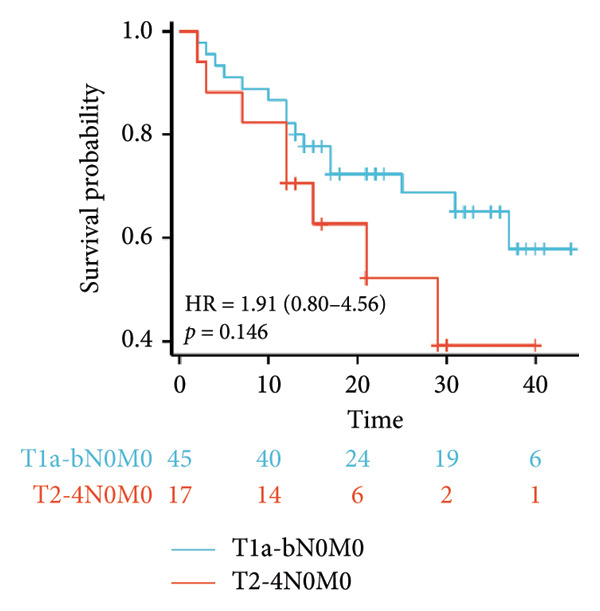
(f)

Figure 4Analysis of the impacts of liver function reserve on overall survival (OS). (a) Kaplan–Meier analysis of OS stratified by Child–Pugh score: < 9 vs. ≥ 9; (b) Kaplan–Meier analysis of OS stratified by ALBI grade: ≤ −1.39 vs. > −1.39; (c) Kaplan–Meier analysis of OS stratified by MELD score: < 14 vs. ≥ 14. ALBI, albumin–bilirubin; MELD, model for end‐stage liver disease.

**Figure:**
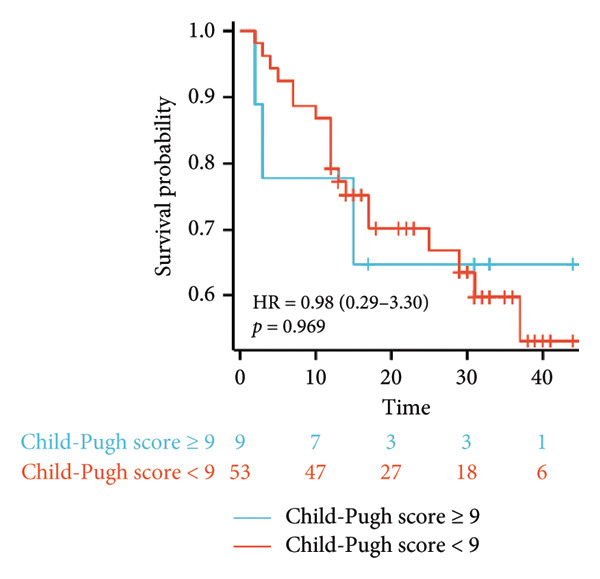
(a)

**Figure:**
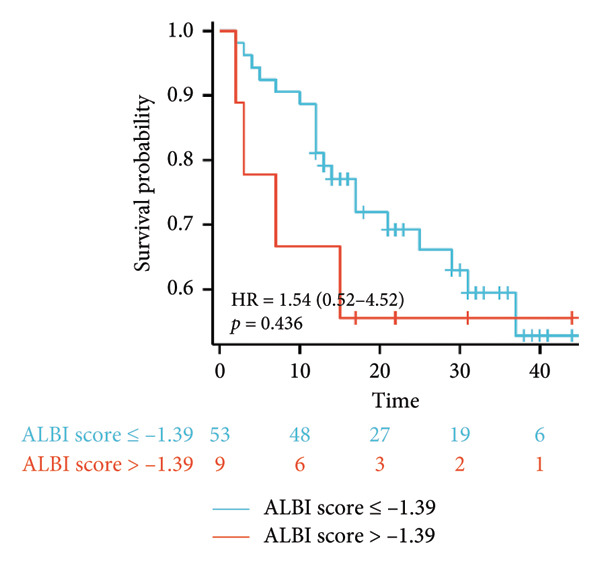
(b)

**Figure:**
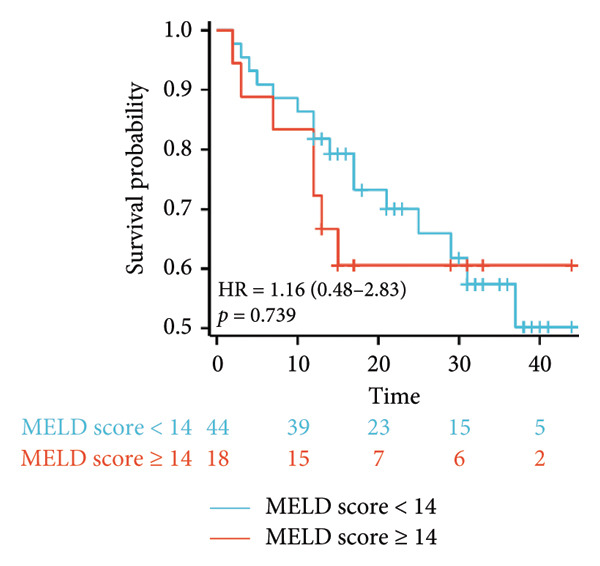
(c)

**Figure 7:**
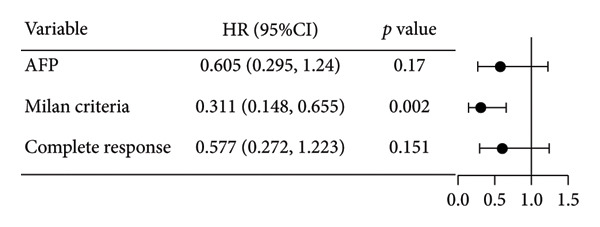
Forest plot of impacts of the clinical factors used in the univariate analysis on overall survival (OS). HR, hazards ratio; CI, confidence interval; AFP, alpha‐fetoprotein.

Initial univariate analysis showed that AFP levels, CR, Milan criteria, and BCLC stage were all significant predictors of tumor progression (Figure 6). The HRs and 95% CIs were as follows: HR = 0.48, 95% CI (0.25,0.92), *p* = 0.028; HR = 0.39, 95% CI (0.20,0.77), *p* = 0.006; HR = 0.30, 95% CI (0.13,0.68), *p* = 0.004; and HR = 0.27, 95% CI (0.13,0.56), *p* < 0.001. Multivariable Cox regression analysis confirmed that the Milan criteria were an independent predictive factor for reduced tumor progression (Figure 7).

Figure 6Analysis of the impacts of tumor characteristics on progress‐free survival (PFS). (a) Kaplan–Meier analysis of PFS stratified by AFP: ≥ 400 vs. < 400; (b) Kaplan–Meier analysis of PFS stratified by treatment response: complete response vs. incomplete response; (c) Kaplan–Meier analysis of PFS stratified by BCLC stage: BCLC 0‐A vs. BCLC B‐C. (d) Kaplan–Meier analysis of PFS stratified by Milan criteria: within Milan criteria vs. beyond Milan criteria. AFP, alpha‐fetoprotein; BCLC, Barcelona Clinic Liver Cancer.

**Figure:**
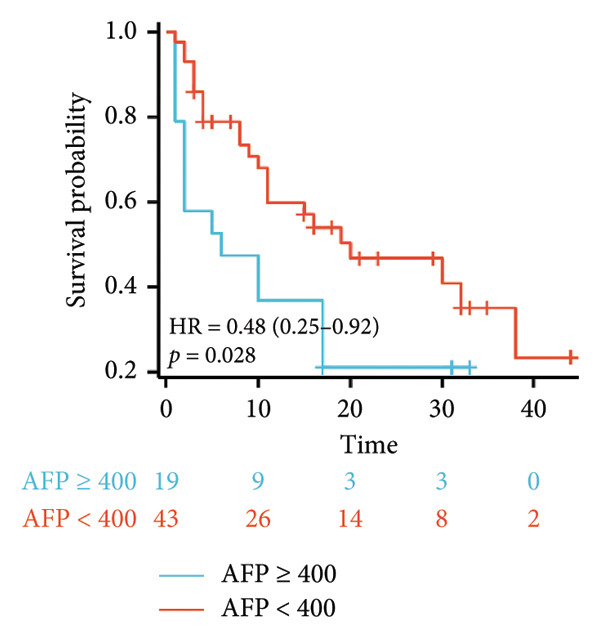
(a)

**Figure:**
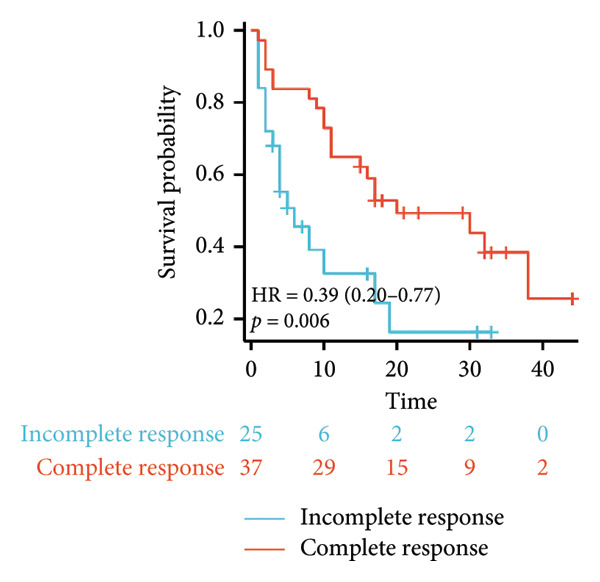
(b)

**Figure:**
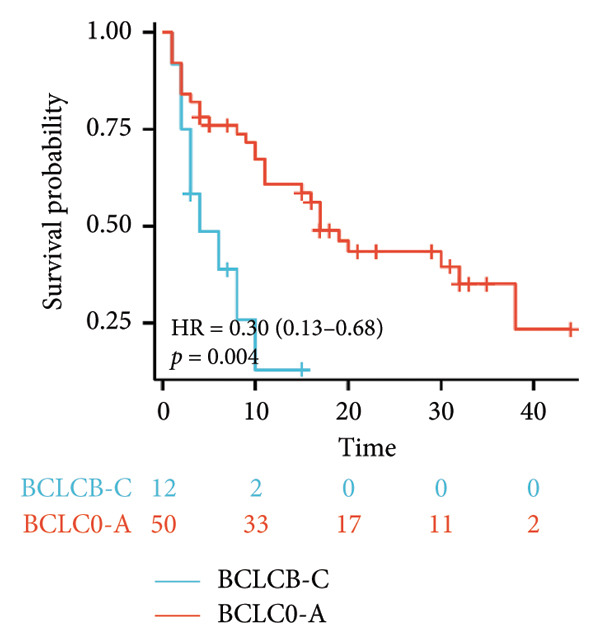
(c)

**Figure:**
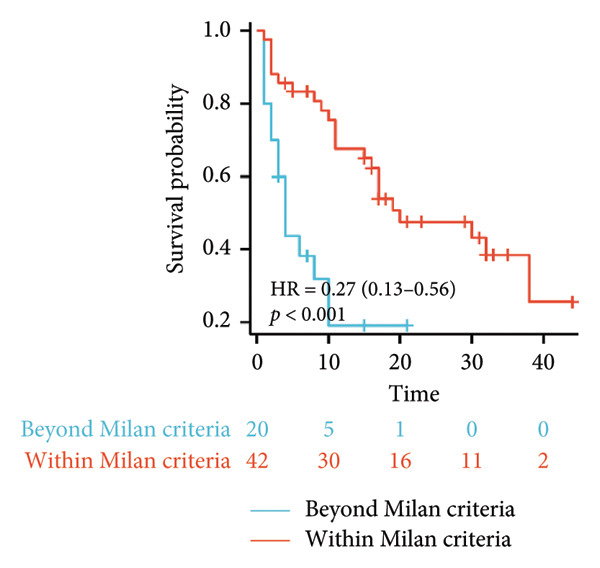
(d)

## 4. Discussion

In China, most HCC patients have liver cirrhosis, with its severity affecting treatment and survival more than HCC stage. Guidelines and registration studies on HCC treatments mainly focus on those with compensated cirrhosis, but many HCC patients with decompensated cirrhosis have diverse clinical profiles and limited treatment options. The OS of patients with advanced‐stage disease remains poor, with a median of only 3.4 months without treatment [22]. The treatment for HCC should be customized for this vulnerable group to optimize survival rates and reduce liver damage risk. LTA is a low‐risk, minimally invasive procedure that may extend OS in early‐stage HCC patients unfit for surgery and can act as a temporary solution before liver transplant [23]. LTA is as effective as surgery for HCC < 5 cm and has become a curative approach [24–27]. However, evidence supporting the advantages of locoregional therapies for individuals with HCC and decompensated cirrhosis is still scarce [28, 29]. Moreover, a study found no survival advantage for LTA in HCC and decompensated cirrhosis patients, as liver function declined faster than HCC progression [10]. The impact of LTA on OS in decompensated cirrhosis remains debated, but selected patients in specialized centers may benefit from LTA in clinical practice.

Given the widespread clinical use of MWA in China, this study evaluated its effectiveness and safety in individuals suffering from decompensated cirrhosis. An examination of data from 62 individuals diagnosed with HCC and decompensated cirrhosis demonstrated the feasibility and safety of MWA, with an average OS of 21.92 months, which exceeded the reported median survival of untreated advanced liver cirrhosis and HCC patients [22, 30]. MWA was relatively safe for patients with decompensated cirrhosis. Only 5 patients experienced minor postoperative complications (abdominal infection), and no Grade 4 or 5 AEs were observed. The postablation increases in ALT, AST, TBIL, and PT levels as well as C‐P, ALBI, and MELD scores improved with symptomatic treatment. However, pre‐MWA liver function reserves as measured by C‐P, ALBI, and MELD scores were not significantly associated with survival in this specific patient population. This is a particularly intriguing result, as liver function is typically a cornerstone of prognostic assessment in HCC. A plausible explanation for this discrepancy is the inherent homogeneity of our cohort, which exclusively comprised patients with decompensated cirrhosis, thereby creating a “floor effect” where the variation in liver function was insufficient to demonstrate a significant differential impact on survival.

The overall safety profile of MWA is demonstrated by its low complication rate, acceptable postprocedural changes in liver function (e.g., controllable hepatic decompensation rates in the MWA group), and survival benefits. A comparative study of MWA versus laparoscopic resection (LR) in patients with clinically significant portal hypertension found that MWA resulted in significantly lower rates of postoperative hepatic decompensation and fewer complications than LR, while achieving comparable 5‐year OS rates [31]. This supports both the short‐ and long‐term safety of MWA. The transient post‐MWA rise in C‐P, ALBI, and MELD scores does not inherently compromise safety. However, studies confirm that higher baseline MELD scores significantly increase the risk of postablation liver function deterioration and adverse outcomes [32, 33]. This transient increase in MELD scores underscores the critical importance of patient selection. We recommend prioritizing patients with lower MELD scores or implementing preoperative liver function optimization and reduced ablation volumes for high‐MELD candidates. Further studies should include stratified analyses of high‐MELD subgroups to refine patient selection criteria.

Although evidence in clinical practice suggests that some patients can benefit from MWA treatment, patients with decompensated cirrhosis have poor tolerance and are not recommended to undergo MWA. Therefore, screening populations that can benefit from MWA treatment is crucial. Most participants had HBV‐related cirrhosis, and survival analysis indicated no significant correlation between cirrhosis etiology, HBV DNA levels, and prognosis. Our results affirmed that tumor burden and treatment response were the paramount drivers of survival in this challenging patient group. The observation that achieving a CR was the strongest independent predictor of improved survival on multivariable analysis is both expected and highly consequential. It underscores that even in patients with advanced liver disease, successful local control of the tumor can significantly alter the natural history of HCC and confer a substantial survival benefit. This conclusion is further reinforced by the consistent significance of established tumor staging criteria. Both the Milan criteria and BCLC 0‐A stage were strongly associated with improved survival and PFS on univariate analysis. The multivariable analysis for PFS confirmed that the Milan criteria were an independent predictive factor for reduced tumor progression. Similarly, a smaller tumor diameter (< 3 cm) was a significant favorable factor, which is consistent with the technical advantages of thermal ablation in achieving adequate ablation margins for smaller lesions.

The LTP rate observed in our study was 10.81%, with a mean LTP‐free time of 24.70 months. This LTP rate falls within the acceptable range reported in the literature for MWA of HCC [34, 35]. This favorable outcome underscores the technical efficacy of MWA in achieving robust local tumor control, even within a cohort of patients with decompensated cirrhosis who pose significant treatment challenges. The relatively prolonged LTP‐free time further suggests that successful ablation can provide a meaningful period of disease control for these patients. During follow‐up, most patients safely underwent repeat locoregional therapy (ablation and TACE) for recurrent HCC, suggesting the feasibility of repeated MWA to improve outcomes.

Our findings indicate that MWA is a safe and effective option for treating patients with decompensated cirrhosis complicated by HCC. In addition, thermal ablation may aid in bleeding control, benefiting patients with hepatic dysfunction and coagulation disorders. This study has some limitations. First, the inherent biases of retrospective studies must be acknowledged. For example, the lack of randomization may lead to confounding factors that could affect the results; there may be selection bias, as the included patients may not represent the broader population. Second, because this is a single‐center, HBV‐dominant population study, the results may not extend to HCV/nonalcoholic steatohepatitis (NASH) or non‐Asian settings. Third, the small sample size of the study limits its reliability and generalizability, suggesting that the findings may not accurately reflect the larger population. Therefore, it is essential to interpret the results with caution and to conduct future research involving larger samples to validate these findings. Fourth, although the median follow‐up duration aligns with the expected lifespan of the study population, a longer follow‐up would provide more reliable survival data. Finally, the lack of a control group weakens the conclusions. Comparing MWA with other local regional therapies (e.g., TACE or radiofrequency ablation) would strengthen the conclusions. Future prospective, multicenter, randomized controlled trials or head‐to‐head comparisons with other therapies are needed to validate the findings. If high‐quality evidence confirms the survival benefits of MWA in patients with decompensated liver cirrhosis, this would support its formal inclusion in future clinical guidelines as a therapeutic option, thereby enhancing patient prognosis and expanding the management landscape for this complex patient cohort.

## 5. Conclusion

Our study results provide evidence that MWA is safe and effective in patients with decompensated cirrhosis complicated by liver cancer. Patients with decompensated cirrhosis who have early‐stage HCC (within Milan criteria, BCLC 0‐A) and are likely to achieve a CR after MWA derive the greatest survival benefit.

## Ethics Statement

This study was conducted in accordance with the Declaration of Helsinki. This retrospective study was reviewed and approved by the Ethics Committee of the First Affiliated Hospital, Anhui Medical University (Hefei, China), with the approval number: PJ2022‐13–41, dated November 10, 2022. Written informed consent was obtained from the participants.

## Consent

Please see the Ethics Statement.

## Disclosure

All authors approved the final version of the manuscript.

## Conflicts of Interest

The authors declare no conflicts of interest.

## Author Contributions

Jihua Xue, Yuting Gu, and Yufeng Gao conceived and designed the study; Jihua Xue, Yuting Gu, Ji Li, Junfei Zhang, Tingting Bian, Zhongsong Zhou, and Yufeng Gao collected the data; Jihua Xue, Yuting Gu, Ji Li, Junfei Zhang, Tingting Bian, Zhongsong Zhou, and Yufeng Gao analyzed and interpreted the data; Jihua Xue and Yuting Gu wrote the manuscript; Jihua Xue, Yuting Gu, Ji Li, Junfei Zhang, Tingting Bian, Zhongsong Zhou, and Yufeng Gao provided critical revisions that are important for the intellectual content. Jihua Xue and Yuting Gu contributed equally to this work and should be regarded as co‐first authors.

## Funding

The work was supported by the States S&T Projects of 13th Five Year (2018ZX10302206‐003‐009), Natural Science Foundation of Education Department of Anhui Province (2022AH040160), and Anhui Province Clinical Medical Research Transformation Project (202304295107020040).

## Supporting Information

Supporting Figure 1: Analysis of the impacts of complete response on overall survival (OS). (A) Kaplan–Meier analysis of OS stratified by treatment response: complete response vs. incomplete response; (B) Kaplan–Meier analysis of OS with 1 month subtracted stratified by treatment response: complete response vs. incomplete response.

## Supporting information


**Supporting Information** Additional supporting information can be found online in the Supporting Information section.

## Data Availability

The datasets used and/or analyzed during the current study are available from the corresponding authors on reasonable request.
