# Supplementary figures and images for: Efficacy and Safety of Microwave Ablation in Patients With Hepatocellular Carcinoma With Decompensated Liver Cirrhosis: A Retrospective Study
Source: Can J Gastroenterol Hepatol. 2025 Nov 12;2025:4859487. doi: 10.1155/cjgh/4859487 (PMC12611878; doi:10.1155/cjgh/4859487)

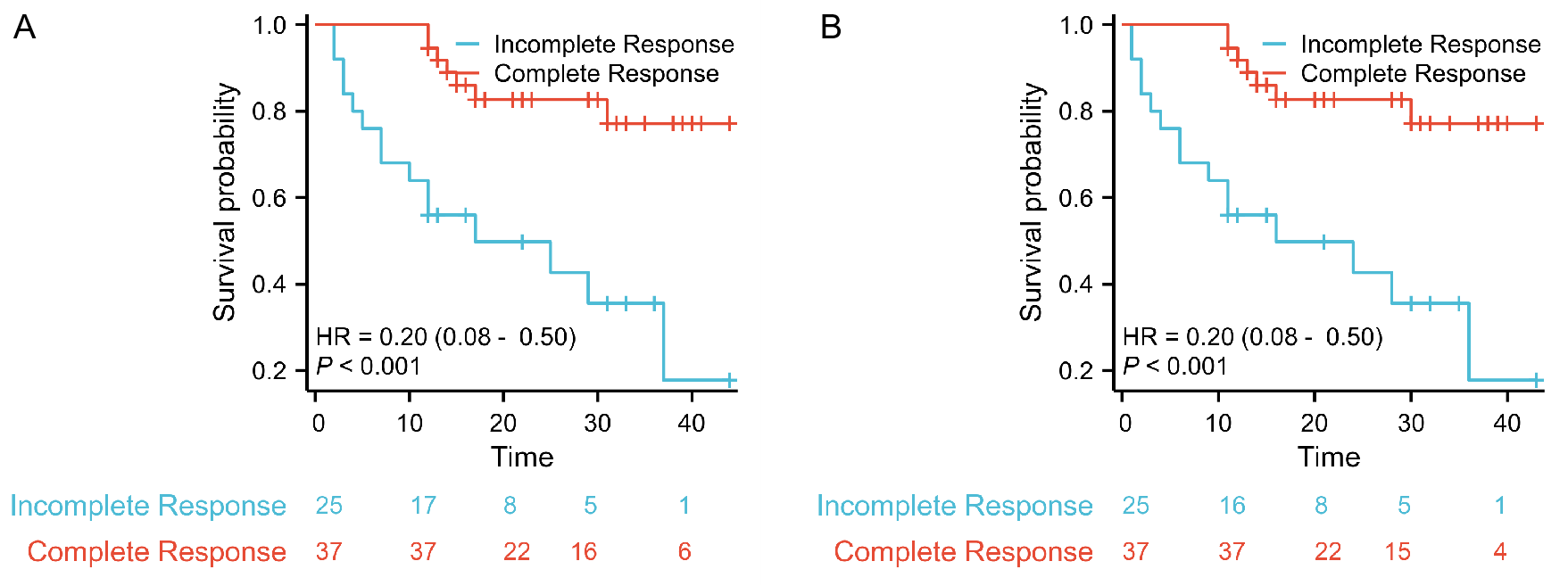

Supplement: Supplementary file 1 — Supporting Information Additional supporting information can be found online in the Supporting Information section. [file CJGH-2025-4859487-s001.tif]
